# Molecular epidemiology and genomic features of *Bordetella parapertussis* in Shanghai, China, 2017–2022

**DOI:** 10.3389/fmicb.2024.1428766

**Published:** 2024-07-09

**Authors:** Pan Fu, Yijia Li, Jie Qin, Li Xie, Chao Yang, Chuanqing Wang

**Affiliations:** ^1^Laboratory of Microbiology, Department of Clinical Laboratory, Children’s Hospital of Fudan University, National Children's Medical Center, Shanghai, China; ^2^Nosocomial Infection Control Department, Children’s Hospital of Fudan University, National Children's Medical Center, Shanghai, China; ^3^CAS Key Laboratory of Molecular Virology and Immunology, The Center for Microbes, Development and Health, Shanghai Institute of Immunity and Infection, Chinese Academy of Sciences, Shanghai, China

**Keywords:** *Bordetella parapertussis*, MT6, MT4, children, China

## Abstract

**Background:**

Pertussis is a highly contagious respiratory illness mainly caused by *Bordetella pertussis* (BP). *Bordetella parapertussis* (BPP) can induce symptoms compatible with pertussis, but has been underdiagnosed and underreported. The current pertussis vaccines offer low protection against BPP. Herein, we aim to reveal the epidemiology and genomic evolution of BPP in Shanghai, China.

**Methods:**

Children diagnosed with BPP infection from January 2017 to December 2022 in Shanghai, China were enrolled. We performed antimicrobial susceptibility testing (AST), multiple locus variable-number tandem repeat analysis (MLVA), and whole genome sequencing (WGS) analysis. A total of 260 international BPP genomes were chosen for comparison to investigate the genomic diversity and phylogenetic characteristics of Chinese strains within a global context.

**Results:**

Sixty patients were diagnosed with BPP infection by culture, with the positive ratio of 3.5‰ (60/17337) for BPP in nasopharyngeal swap samples. The average age of patients was 4.5 ± 0.3 years. BPPs contained four MLVA types including MT6 (65.0%), MT4 (26.7%), untype-1 (6.7%) and MT5 (1.7%), and none of strains showed resistance to macrolides. All strains carried virulence genotype of *ptxP37/ptxA13/ptxB3/ptxC3/ptxD3/ptxE3/fim2-2/fim3-10*. MT4 and MT5 strains carried *prn54*, whereas MT6 and untype-1 BPPs expressed *prn101*. We identified two outbreaks after 2020 caused by MT4 and MT6 strains, each corresponding to distinct WGS-based phylogenetic lineages. The MT4-lineage is estimated to have originated around 1991 and has since spread globally, being introduced to China between 2005 and 2010. In contrast, the MT6-lineage was exclusively identified in China and is inferred to have originated around 2002.

**Conclusion:**

We revealed the genomic diversity of BPPs circulating in Shanghai, China, and reported the outbreaks of MT6 and MT4 BPPs after 2020. This is the first report on the emergence and regional outbreak of MT6 BPPs in the world, indicating that continuous surveillance on BPPs are thus required.

## Introduction

Whooping cough (pertussis) is a highly contagious respiratory disease of humans, which is mainly caused by *Bordetella pertussis* (BP; Feng et al., 2021; Gorgojo et al., 2023). Compared to BP, *B. parapertussis* (BPP) causes a milder whooping cough-like syndrome and is responsible for a smaller proportion (2%~20%) of pertussis (Toubiana et al., 2019). However, BPP infection has been poorly recognized in the world. Pertussis was previously thought to mainly occur in infants (Fu et al., 2019). However, more studies reveal that the prevalence and re-emergence of pertussis has been increasing in older children, adolescents and adults, making a great public threaten in the world (Moore et al., 2019; Zhang et al., 2022).

There are many differences between BBP and BP. For example, BPP lacks the production of the pertussis toxin (Ptx) due to a mutation in the promoter region of the genes encoding this toxin (Arico and Rappuoli, 1987). The World Health Organization (WHO) recommended two types of approach to diagnosis, including direct diagnosis [culture, real-time polymerase chain reaction (RT-PCR)] and indirect diagnosis (serology). These two species can be distinguished based on a number of biochemical characteristics: BPP grow faster and appear grayish; the oxidase test was positive in BP but negative in BPP, etc. Moreover, a series of targets including IS481, IS1001, IS1002, etc. were used to distinguish different *Bordetella* species. For example, IS1001 which presented in all BPPs but was absent in BPs, was widely used to identify BPP (Riffelmann et al., 2005).

BPP might vary from an unrecognized infection to a mild illness or typical pertussis presentation; it is increasingly recognized and reported to public health agencies (Liko et al., 2017). Pertussis vaccines are produced as combination vaccines with diphtheria and tetanus toxoids. In China, a routine immunization schedule of diphtheria, tetanus, whole-cell pertussis vaccine (DTwP) was implemented in the 1960s. Starting in 2005, both DTwP and diphtheria-tetanus-acellular pertussis vaccine (DTaP) were used in China, with DTaP gradually replacing DTwP by 2010 (Wu et al., 2023). Although DTap vaccine significantly reduced the incidence of pertussis, many studies have shown that pertussis vaccination is irrelevant to or just partially protect against BPP infection (Liko et al., 2017). The rodent model showed that aP vaccination, by priming the host response against BP clearance, confers an advantage to BPP by interfering with optimal immune clearance and resulting in increased lung colony-forming units (Long et al., 2010).

Until now, a series of studies on BP strains were reported in China, including Zhejiang province (Lin et al., 2022), Shanghai (Fu et al., 2023b), Shenzhen (Wu et al., 2021), and Beijing (Zhou et al., 2024). However, systematic studies or reports on BPP strains are very scarce in the world. National surveillance on BPP strains are largely lacking in China. In this study, we performed a continuous surveillance on *Bordertella* spp. based on culture, and collected a total of 60 BPP strains from January 2017 to December 2022 in Shanghai, China. We systematically analyzed the clinical and epidemiology features, the antimicrobial resistance (AMR) profiles, and the genomic evolution of those strains.

## Materials and methods

### Enrollment of pertussis cases

From January 2017 to December 2022, there were 740 children diagnosed as pertussis by bacterial culture in Shanghai, China. Their nasopharyngeal swab (NP) samples were collected for *Bordetella* spp. culture and antimicrobial resistance testing. Their basic information, clinical diagnosis, and X-ray imaging were collected based on the electronic medical records. The laboratory testing results were collected and analyzed in this study, including white blood cell counts (WBC, ×10^9^/L) and C-reactive protein (CRP, mg/L). All data collection and analysis were anonymous. This study was approved by the Ethics Committee of the Children’s Hospital of Fudan University (no. 2022-66).

### Culture and antimicrobial susceptibility testing of BPP strains

NP samples were delivered to clinical microbiology laboratory and immediately spread onto charcoal agar (OXOID, United Kingdom) plates supplemented with 10% defibrinated sheep blood and cephalexin (40 mg/L). The plates were incubated in a humidified incubator at 35°C for 3 to 5 days. Different *Bordetella* species were verified by Gram staining, biochemical tests, and Matrix assisted laser desorption ionization-time of flight mass spectrometry (MALDI-TOF MS, Bruker, Germany).

The BPP isolates were suspended equivalent to a 0.5 McFarland standard and inoculated onto charcoal agar containing 10% sheep blood without cephalexin. The minimum inhibitory concentrations of four antimicrobial agents, including erythromycin, azithromycin, clarithromycin, and sulfamethoxazole/trimethoprim, were determined by the E-test after 72 h of incubation at 35°C. The standardized interpretation criteria are based on our previous report (Fu et al., 2019).

### Whole genome sequencing and analysis

Genomic DNA of BPP strains were extracted using QIAamp DNA mini kit (QIAGEN) and whole-genome sequencing were performed on Illumina NovaSeq platform. Sequencing data were analyzed as previously described (Yang et al., 2022; Fu et al., 2023b). Briefly, species identification was performed using Kraken 2 based on sequencing data (Lu et al., 2022).^1^ Genome assembly was performed using shovill pipeline. The genome characteristics of newly sequenced data were calculated using Quast v5.0.2 (Gurevich et al., 2013). The prevalence of insertion sequence (IS) IS1001 were detected by searching against assembled genome sequences using BLASTN. It was considered present if the BLASTN hit coverage and identity were at least 90%. Core-genome single-nucleotide-polymorphisms (SNPs) were identified using the Snippy pipeline,^2^ with strain 12822 [accession number: (NC_002928.3)] as the reference genome. Maximum-likelihood phylogenetic trees were constructed using RAxML-NG based on core-genome SNPs (Kozlov et al., 2019). The maximum-likelihood tree was rooted using the midpoint method. The dated phylogenetic trees were automatically rooted based on temporal signal using a root-to-tip linear regression with BactDating (Didelot et al., 2018). New sequencing data have been deposited in NCBI Sequence Read Archive (SRA) under accession number PRJNA1060880.

A total of 260 publicly available international genomes were downloaded from NCBI GenBank or SRA database, with accession numbers listed in the appendix (Supplementary Table S1). The international BPP strains included France (118), USA (91), Spain (29), Germany (3), Austria (2), United Kingdom (1), Australia (1), Japan (1), Iran (1), and unknow (13).

### Multiple locus variable-number tandem repeat analysis, multilocus sequence typing, and *Bordetella* spp. virulence genotyping analysis

Genomic DNA of BPP isolates was prepared by a QIAamp DNA mini kit (QIAGEN). Multiple locus variable-number tandem repeat analysis (MLVA) was performed following the procedures according to the report of Kamachi et al. (2019). Four loci (VNTR4, VNTR13, VNTR14, and VNTR15) were amplified by PCR. The number of repeats at each VNTR locus was calculated from the DNA fragment length. The assignment of an MLVA type (MT) was based on the combination of repeat counts for VNTR4, VNTR13, VNTR14, and VNTR15 according to previous reports (Kamachi et al., 2019).

Multilocus sequence typing (MLST) were analyzed by seven housekeeping genes (*adk*, *fumC*, *glyA*, *tyrB*, *icd*, *pepA*, and *pgm*). BPP genomes data were matched on the website.^3^ The alleles at each of the seven loci defined the allelic profile or sequence type (ST).

Assembled BPP genome sequences were used for virulence genotyping by searching against BIGSdb-Pasteur genomic platform for *Bordetella*.20. The virulence-related genes included pertussis toxin (PTX) promoter (*ptxp*), five *ptx* genes (*ptxA, ptxB, ptxC, ptxD*, *ptxE*), pertactin (*prn*), filamentous hemagglutinin B (*fhaB*), and fimbrial proteins (*fim2, fim3*).

### Statistical analysis

All statistical analyses were performed using the GraphPad Prism software version 8.0. The *t* test and Bonferroni correction were performed to compare the differences of clinical characteristics and laboratory testing results between two groups. A *p-*value of less than 0.05 was considered statistically significant.

## Results

### Distributions and detection of BPP cases from 2017 to 2022

**Figure 1:**
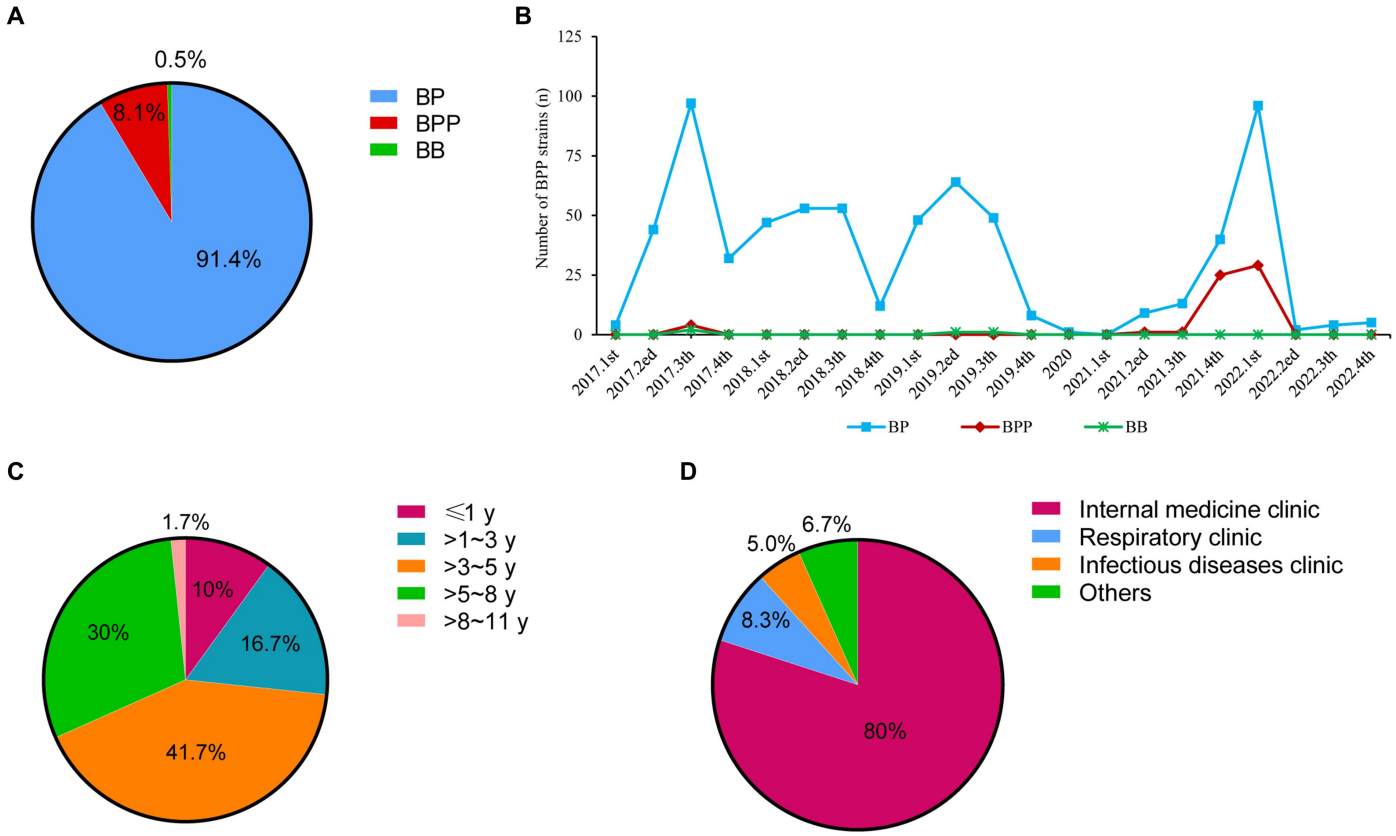
Distributions of *Bordetella* species and basic information of BPP infection cases from 2017 to 2022. **(A)** Distributions of different *Bordetella* species; **(B)** Detection ratios of three *Bordetella* species from 2017 to 2022; **(C)** Age distributions of BPP infection cases; **(D)** Department distributions of BPP infection cases. BP, *Bordetella pertussis*; BPP, *Bordetella parapertussis*; BB, *Bordetella bronchitis*.

As shown in Figure 1A, there were 740 children diagnosed as pertussis by bacterial culture, and 91.4% (676 patients), 8.1% (60 patients) and 0.5% (4 patients) of the pertussis were caused by BP, BPP, and *Bordetella bronchitis* (BB), respectively.

BP strains were continuously isolated from 2017 to 2019 (ranging from 4 strains to 97 strains per quarter), but only one BP strain was collected in 2020. Notably, BP infection was re-emerged from 2021 to 2022, and there were two peaks of BP infection at 4th quarter of 2021 (40 strains) and 1st quarter of 2022 (96 strains). Compared to BP cases, BPP infection was quite scarce before 2021. Only four BPP strains were identified at 3rd quarter of 2017. Concurrent with the BP re-emergence time, BPP infection had outbreaks at 4th quarter of 2021 (25 strains) and 1st quarter of 2022 (29 strains; Figure 1B).

Among 17,337 patients who received nasopharyngeal swap samples culture, there were 60 children diagnosed with BPP infection, with the positive ratio of 3.5‰ (60/17337). The average ages were (4.5 ± 0.3) years old. The infants (≤1 year), toddler (>1~3 years), preschool (>3~5 years), school age (>5~8 years) and adolescents (>8~11 years) accounted for 10% (6), 16.7% (10), 41.7% (25), 30% (18), and 1.7% (1), respectively (Figure 1C). Most of patients came from the internal medicine clinic (80%, 48; Figure 1D).

### Clinical and laboratory characteristics of BPP cases

As shown in Table 1, most of the patients (76.7%, 46 patients) presented paroxysmal cough and phlegm, and the average cough period was (27.0 ± 4.6) days. Moreover, there were 56.7% (34 patients), 30% (18 patients), 21.7% (13 patients), 13.3% (8 patients) and 6.7% (4 patients) of BPP cases presented rhinorrhea, fever, vomiting, wheezing and spasmodic cough, respectively. We further compared the difference of younger children (1.7 ± 0.3 years old, 2 months to 3 years) and older children (5.6 ± 0.2 years old, >3 years to 11 years). It is noted that younger children presented more severe clinical symptoms than older children, including longer cough periods [(35.3 ± 9.6) days vs. (20.0 ± 3.2) days, *p* = 0.03), vomit (56.3% vs.9.1%, *p* < 0.01), and wheezing (31.3% vs.6.8%, *p* = 0.04].

**Table 1:** Clinical and laboratory characteristics of BPP cases during 2021 to 2022.

Characteristics		Total	Younger children	Older children	*p*-value
		(*n* = 60)	(≤3 years old, *n* = 16)	(>3 years old, *n* = 44)	
Gender	Male	32 (53.3%)	11 (68.8%)	21 (47.7%)	
	Female	28 (46.7%)	5 (31.3%)	23 (52.3%)	0.15
Ages (Years)		4.5 ± 0.3	1.7 ± 0.3	5.6 ± 0.2	**<0.01**
Clinical symptoms	Paroxysmal cough	46 (76.7%)	12 (75.0%)	34 (77.3%)	0.87
	Cough days	27.0 ± 4.6	35.3 ± 9.6	20.0 ± 3.2	**0.03**
	Spasmodic cough	4 (6.7%)	2 (12.5%)	2 (4.5%)	0.58
	Vomit	13 (21.7%)	9 (56.3%)	4 (9.1%)	**<0.01**
	phlegm	46 (76.7%)	13 (81.3%)	33 (75.0%)	0.87
	Rhinorrhea	34 (56.7%)	10 (62.5%)	24 (54.5%)	0.58
	Wheezing	8 (13.3%)	5 (31.3%)	3 (6.8%)	**0.04**
	Fever	18 (30.0%)	5 (31.3%)	13 (29.5%)	0.9
Antimicrobial usage	Macrolides	30 (50%)	9 (56.3%)	19 (43.2%)	0.37
	Cephalosporins	24 (40%)	9 (56.3%)	15 (34.1%)	0.12
Antimicrobial resistant ratios	Macrolides	0 (0%)	0 (0%)	0 (0%)	>0.99
	Sulfamethoxazole/trimethoprim	0 (0%)	0 (0%)	0 (0%)	>0.99
X-ray	Bronchitis	27 (45.0%)	11 (68.8%)	16 (36.4%)	0.54
	Bronchopneumonia	8 (13.3%)	3 (18.8%)	5 (11.4%)	0.75
	Pneumonia	3 (5.0%)	0 (0%)	3 (6.8%)	0.56
Laboratory testing	WBC	11.4 ± 0.4	12.2 ± 0.9	11.2 ± 0.5	0.28
	Abnormal CRP	14 (23.3%)	4 (25.0%)	10 (22.7%)	0.97

White blood cell counts (WBC, 10^9^/L), C-reactive protein (CRP, mg/L), Abnormal CRP was defined as CRP ≥ 8 mg/L. Statistical analyses were performed with GraphPad Prism 8.00. The t test and Bonferroni correction were performed to compare the differences of clinical characteristics and laboratory testing between two groups. A *p*-value of less than 0.05 was considered statistically significant, and was bolden in the table.

There were 50% (30 patients) and 40% (24 patients) of BPP cases treated by macrolides and cephalosporins, respectively. However, no BPP strains isolated from the patients showed resistance to macrolides, and all strains presented sensitive to sulfamethoxazole/trimethoprim. The chest X-ray of BPP cases presented either bronchitis (45.0%, 27), bronchopneumonia (13.3%, 8) or pneumonia (5.0%, 3), and there was no difference between younger children and older children. WBC was slightly increased to (11.4 ± 0.4) × 10^9^/L, and 23.3% (14) of patients presented abnormal CRP (>8 mg/L; Table 1).

### Prevalence of different BPP types from 2017 to 2022

**Figure 2:**
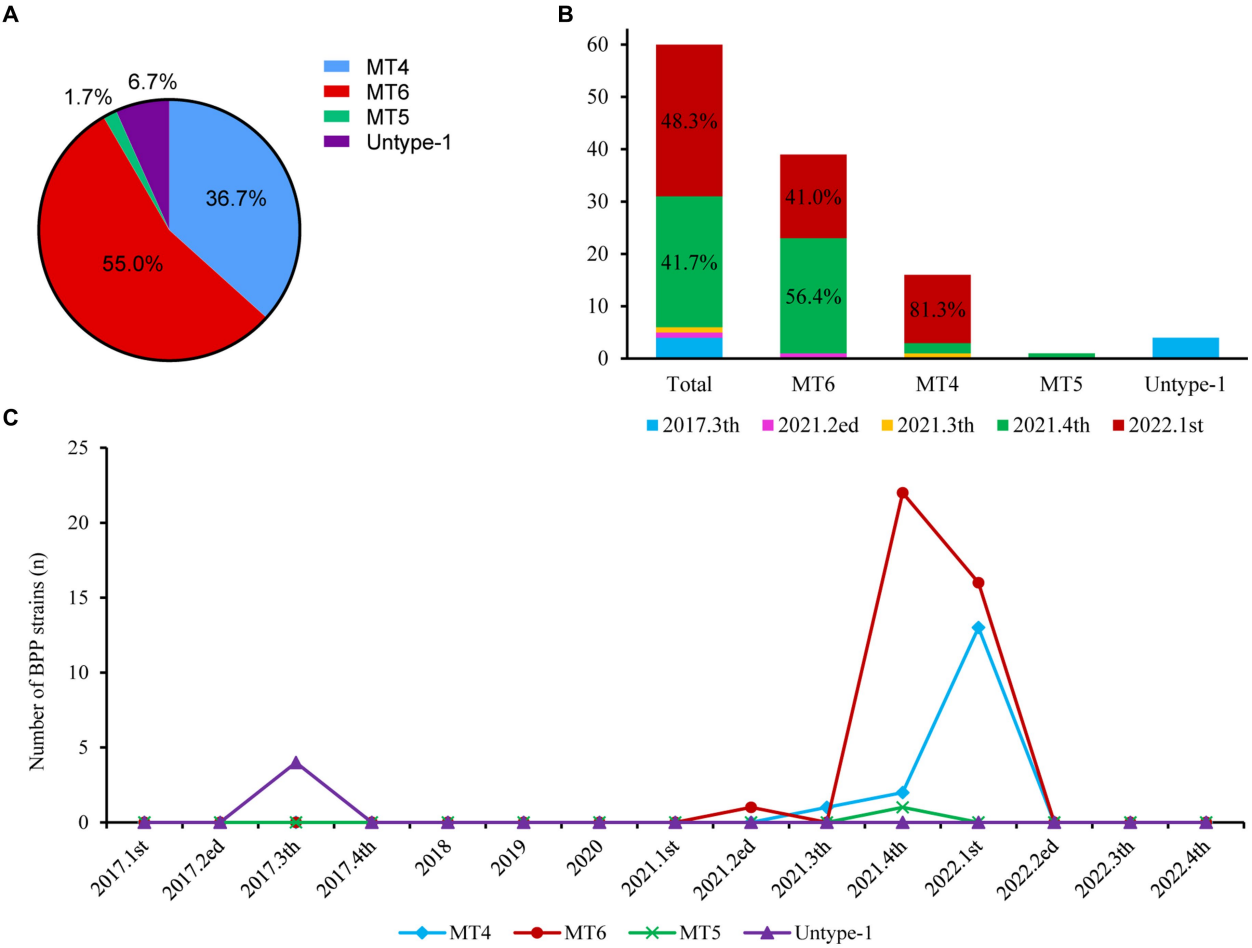
Prevalence of different BPP subtypes from 2017 to 2022. **(A)**. MT subtypes of all BPPs; **(B)**. Distributions of different MLVA types over time; **(C)**. Detection of different BPP subtypes per quarter from 2017 to 2022.

All 60 BPP strains belonged to one MLST type 19 (ST19, 100%), whereas MLVA analysis further revealed four different BPP subtypes in this study. MLVA type 6 (MT6) with the VNTR profiles of 4-5-13-5 was the major subtype (65.0%, 39 strains) of BPPs in this study, followed by MT4 (VNTRs: 3-7-18-4, 26.7%, 16 strains). Other subtype including MT5 (VNTRs: 3-7-21-4) and untype-1 (VNTRs: 4-5-10-4) were less frequently detected, with the ratios of 1.7% (1 strain) and 6.7% (4 strains), respectively (Figure 2A).

MT6 and MT4 were mostly isolated at 4th quarter, 2021 (56.4% and 12.5%) and 1st quarter, 2022 (41.0% and 81.3%), respectively (Figure 2B). There were MT6-BPP outbreaks at 4th quarter, 2021 (22 strains) and 1st quarter, 2022 (16 strains), and MT4-BPP outbreak at 1st quarter, 2022 (13), respectively (Figure 2C).

### Genomic characteristics and evolution of Shanghai BPP strains

For the newly sequenced strains, the average GC content, number of contigs and size of assemblies were 68.17% (68.16–68.17%), 80 (70–92) and 4.72 Mb (4.72–4.73), with an average of 92-fold (87–141) depth for each genome. The marker sequence of BPP, IS1001, was found in all Chinese BPP strains.

**Figure 3:**
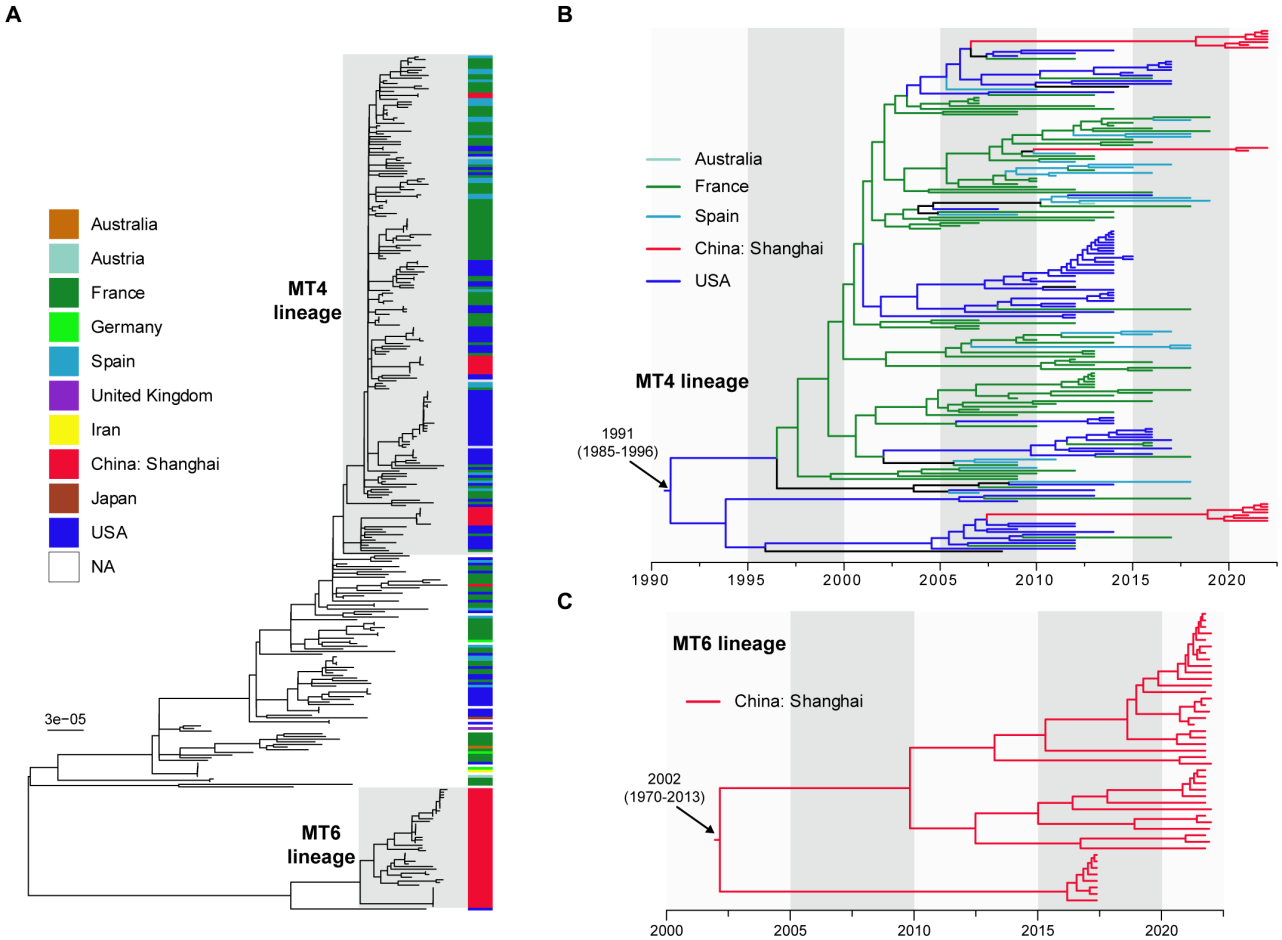
The dated maximum-likelihood phylogenetic tree of 60 Shanghai, China and 260 international BPP strains **(A)**. Different colors presented different geographic areas and red color indicates Shanghai strains. MT4-lineage **(B)** and MT6 lineage **(C)** are enlarged for visualization. NA, not applicable. The maximum-likelihood tree **(A)** was rooted using the midpoint method. The dated phylogenetic trees **(B,C)** were automatically rooted based on temporal signal.

We compared 60 Shanghai BPPs with 260 public genomes of global BPPs to reveal the phylogenetic relationship of those strains. After integrated the MLVA subtypes of 60 BPPs with WGS analysis, we defined two lineages: MT4-lineage and MT6-lineage, corresponding to MT4 and MT6 strains that caused disease outbreaks. As shown in Figure 3A, MT4-lineage isolates in Shanghai were closely related to those isolated from the France, United States, Austria, and Spain. MT6-lineage strains isolated after 2020 in Shanghai were quite different to other strains. Figure 3B showed that MT4-lineage was estimated to have originated at 1991 [95% confidence interval (CI): 1985~1996] probably from USA, and has spread to multiple regions, including Europe (France and Spain), Australia and China. This lineage was inferred to have been introduced to China around 2005 to 2010, much earlier than our first MT4 strain isolated in 2021. MT6-lineage was different to either the MT4-lineage strains or other international strains. Notably, this lineage was estimated to have originated in China at 2002 (95% CI: 1970~2013) and was only identified in Shanghai, China (Figure 3C).

We further identified 74 SNP sites that can be used to distinguish MT4-lineage and MT6-lineage, with alleles that are completely different in the two lineage strains. Among these SNPs, 64 located within gene regions (64 genes, each with one SNP), and an additional 10 SNPs located in intergenic regions (Supplementary Table S3). SNP sites can be used to distinguish MT4- and MT6-lineages, exhibiting alleles that are completely different in the two lineage strains. For example, the SNP at position 130,756 has two alleles: A and C. All MT4-lineage strains carried allele A, while all MT6-lineage strains carried allele C.

In this study, BPPs expressed a series of *Bordetella* virulence factors, with the profiles of *ptxP37/ptxA13/ptxB3/ptxC3/ptxD3/ptxE3/ fim2-2/fim3-10*. All of MT4 and MT5 strains carried *prn54*, whereas MT6 and untype-1 BPs expressed *prn101* (Supplementary Table S2). The *fhaB* genes included four different alleles: *fhaB-45* (48.3%), *fhaB-3* (18.3%), *fhaB-22* (18.3%), and *fhaB-44* (15.1%), all of which were not related to any MT subtypes.

We further compared the clinical and laboratory characteristics of MT4-lineage and MT6-lineage BPPs (Supplementary Table S4). There were no big differences between those two BPP lineages in Shanghai (*p* > 0.05), including the clinical symptoms, the inflammatory factors, X-rays, antimicrobial treatment history and AMR profiles. However, MT6-lineage BPP cases presented longer cough periods of (31.2 ± 5.6) days, which was two or more times as long as MT4-lineage (14.2 ± 2.7 days, *p* = 0.04).

## Discussion

BPP can cause whooping cough in human, but the epidemiology of respiratory illness caused by BPP strains has been poorly recognized in the world (Mastrantonio et al., 1998). Herein, we collected a total of 60 BPPs from 2017 to 2022 in Shanghai, China, and systematically analyzed the clinical and epidemiologic features and the genomic evolution of those strains. BPP accounted for 8.1% of whooping cough in this study, and caused more severe clinical symptoms in younger children (0~3 year). BPPs circulating in Shanghai contained four different MT types. It is noted that there was the outbreak of BPP infection after 2020, and the major MLVA types were MT4 and MT6. These two subtypes have evolved independently: MT4-lineage was highly homogeneous to international BPPs which were estimated to originate at USA and introduced to China around 2005 to 2010, whereas MT6-lineage was estimated to originate in China and was only identified in Shanghai, China.

After aP vaccine replaced wP vaccine by 2010 in China, there are two types of DTaP formulations licensed in China: one is the two-component DTaP containing Ptx and Fha, and another is the three-component DTaP containing Ptx, Fha and Prn. The current vaccine in Shanghai contains Ptx and Fha. However, BP vaccines fail to or partially induce protection against BPPs and the incidence of this species has been rising over the years (Long et al., 2010; Liko et al., 2017). BPP has been circulating worldwide and causes outbreaks despite high pertussis vaccine coverage of young children. Unlike BP infections which were primarily identified in infants before 2020 in Shanghai, China (Fu et al., 2019, 2023b), the average ages of BPP cases were (4.5 ± 0.3) years, and only 10% patients aged less than 1-year-old. The average cough period of BPP infection was much longer than our previous report of BP infection (27.0 ± 4.6 days vs. 15.5 ± 0.8 days; Fu et al., 2023a). Moreover, younger children (0–3 years) presented more severe clinical symptoms than those aged more than 3-years-old. Therefore, we must pay attention to BPP infections among children, especially the younger children.

Previously, erythromycin has been the mainstay of antibiotic therapy for pertussis as it decreases the transmission of infection and ameliorates symptoms particularly in younger and more severely affected infants (Mortensen and Rodgers, 2000). However, after the erythromycin-resistant BPs in China was firstly isolated in Shandong Province, China in 2011, more macrolides-resistant BP (MRBP) strains were reported in China, making macrolides less effective against BP infection (Zhang et al., 2013). Antimicrobials such as macrolides and sulfamethoxazole/trimethoprim recommended for BP infection have also been used for treating and preventing BPP infection (Mortensen and Rodgers, 2000). This study revealed a high proportion of macrolides treatment (50%) against BPP infection, but none of the BPPs were resistant to macrolides. This is quite different with high macrolides resistance of BPs in China (Feng et al., 2021; Wu et al., 2022; Fu et al., 2023b), indicating that macrolides are still effective against BPP infection. The resistance mechanism in BPs and BPPs are different: The 23S rRNA A2047G mutation is considered the major mechanism of resistance to macrolides in BP strains. However, the macrolides resistance mechanism in BPP is still unclear. Lately, Fong et al. (2022) reported that the macrolides resistance in BPP was probably related to the upregulation of an efflux pump mechanism, but it still needs further investigation. Therefore, we hypothesize that the macrolides resistance due to A2047G mutation was stable and has the potential spread capability than any other resistance mechanisms.

BPP can caused regional or national outbreaks. For example, Koepke et al. (2015) reported the concurrent outbreak of BPPs and BPs during 2011 to 2012 in Wisconsin, USA, and the BPPs accounted for nearly 6.0% of pertussis cases. In this study, we identified BPP outbreak after 2020 in Shanghai, China, which is greatly consistent with BP re-emergence time after 2020. We hypothesized that the potential re-emerging of BPPs and BPs in this study was related to the suppressed spread or circulation of respiratory pathogens during COVID-19. There were two major MLVA types including MT6 and MT4, the VNTR profiles of MT6 and MT4 were quite heterogenous, revealing the independent spread and genomic evolution of these two subtypes. Recently, Kamachi et al. (2019) constructed the MLVA analysis method of BPP strains, and identified one MT6 strain isolated at 2010 in Taiwan, China, and two MT4 strains isolated at 2010 in Taiwan, China and at 1988 in France, respectively.

BPP and BP share the same virulence factors including Prn, dermonecrotic toxin, Fha and adenylate cyclase (Mastrantonio et al., 1998). However, the Ptx as one of the major virulence factors is only expressed in BP since the *ptx* operon in BPP is dysfunctional (Arico and Rappuoli, 1987). In this study, many of the virulence factors characterized in BP strains are commonly expressed in BPPs. BPPs in Shanghai carried a series of virulence factors, including *ptxP37/ptxA13/ptxB3/ptxC3/ptxD3/ptxE3/fim2-2/fim3-10*. It is noted that although BPPs carry *ptx* genes in the genomes, they cannot express and secrete the pertussis toxin due to mutation in Ptx promoter region. *Prn* allele was diverse among different BPP subtypes: MT4 and MT5 strains all carried *prn54*, whereas MT6 and untype-1 BPs expressed *prn101*, revealing the heterogeneity of virulence genes among different BPP subtypes. Prn-deficient BPs have widely been reported in countries using aP vaccines, such as the United States (85%), Australia (>80%), Sweden (69%), and Italy (55%; Byrne and Slack, 2006; Martin et al., 2015; Zomer et al., 2018; Weigand et al., 2019; Ma et al., 2021). Herein, all BPPs expressed *prn* gene without any mutation or disruption, and none of *prn*-negative BPP was identified. The aP vaccines containing Prn as an immunogen was thought to be the selection pressure for *prn*-negative BP strains (Ma et al., 2021). The current ACVs in Shanghai only contain Ptx and Fha, so we hypothesized that *prn* expression was not influenced by vaccine pressure because the current ACVs used in Shanghai contain no Prn antigen.

We collected 260 international BPPs for comparison, revealing the different genomic characteristics and molecular evolution of MT4-lineage and MT6-lineage. Firstly, 64 SNPs in gene regions and 10 SNPs in intergenic regions were identified among these two lineages. Secondly, the dated phylogenetic trees further revealed evolutionary differences between MT4- and MT6-lineage: MT4-lineage which originated at 1991 from USA was introduced to China around 2005~2010; MT6-lineage presented genomic heterogeneity to any other BPP strains, and was exclusively identified in Shanghai, China. We further compared the clinical and laboratory features of MT4-lineage and MT6-lineage BPPs. Although most of the clinical manifestations and inflammatory factors between these two lineages showed no significant differences, MT6-lineage cases presented the longer cough period (31.2 ± 5.6 days), which was about two or more times as long as MT4-linage cases. Therefore, it is thus important to keep continuous surveillance of MT-6 lineage BPP strains.

In summary, we revealed the genomic diversity and molecular evolution of different BPP subtypes circulating in China, and reported the emergence and outbreak of MT6 and MT4 BPPs after 2020 in Shanghai, China. To the best of our knowledge, it is the first report on the emergence and regional outbreak of MT6-lineage BPP in the world, highlighting that continuous surveillance and effective detection on BPP strains are thus required.

## Data availability statement

The original contributions presented in the study are publicly available. The names of the repository/repositories and accession number(s) can be found in the article/Supplementary material.

## Ethics statement

The studies involving humans were approved by the Ethics Committee of the Children’s Hospital of Fudan University (no. 2022-66). The studies were conducted in accordance with the local legislation and institutional requirements. Written informed consent for participation in this study was provided by the participants’ legal guardians/next of kin.

## Author contributions

PF: Funding acquisition, Supervision, Writing – original draft, Writing – review & editing. YL: Data curation, Investigation, Methodology, Writing – review & editing. JQ: Investigation, Methodology, Writing – review & editing. LX: Methodology, Writing – review & editing. CY: Supervision, Writing – original draft, Writing – review & editing. CW: Supervision, Writing – original draft, Writing – review & editing.

## Footnotes

^1^ https://github.com/tseemann/shovill

^2^ https://github.com/tseemann/snippy

^3^ https://bigsdb.pasteur.fr/bordetella/
